# A Systematic DFT Approach for Studying Mechanisms of Redox Active Enzymes

**DOI:** 10.3389/fchem.2018.00644

**Published:** 2018-12-21

**Authors:** Per E. M. Siegbahn, Margareta R. A. Blomberg

**Affiliations:** Arrhenius Laboratory, Department of Organic Chemistry, Stockholm University, Stockholm, Sweden

**Keywords:** density functional theory, redox reactions, nitrogenase, cytochrome *c* oxidase, exact exchange

## Abstract

When DFT has been applied to study mechanisms of redox processes a common procedure has been to study the results for many different functionals. For redox reactions involving the first row transition metals, this approach has given very different results for different functionals. The conclusion has been that DFT cannot be used for these reactions. In the meantime, results with strong predictability have been generated, most noteworthy for photosystem II, where all DFT predictions have been verified by experiments performed later. In order to obtain these predictive results using DFT, an alternative, systematic approach has been used, where the key differences between the results for different functionals can be rationalized by using a single parameter, rather than using the very large number of differences in the functionals.

## I. Introduction

The results using density functional theory for molecules, have been continuously criticized the past decades. In particular, the results using b3lyp (Becke, 1993) have been shown to have severe errors in several cases. For main group elements, most of these cases were found to be corrected by the addition of dispersion corrections (Schwabe and Grimme, 2007). The cases where dispersion was needed could relatively easily be identified, and the majority of calculations made using b3lyp were not affected by dispersion in a significant way. However, molecules containing transition metals have always been considered particularly difficult to treat, and the use of DFT has continued to be criticized in these cases. DFT using different functionals were shown to give very different results for many redox reactions, in particular for molecules containing first row transition metals. The selection of different functionals for different reactions has become popular, but this approach obviously suffers from user bias, and lack of predictability.

When different functionals are compared, it is important to do this in a systematic way. During the past decade it has been possible to identify the most sensitive parameter for redox enzymes, and this is the amount of exact exchange. By identifying this single parameter, it is possible to get away from the non-systematic approach of testing a large number of functionals, which differ in many irregular ways. The best procedure found is to start with the usual b3lyp functional, which has 20% exact exchange, and decrease this percentage in steps.

A breakthrough for that approach came in the studies of photosystem II (Siegbahn, 2009, 2013). The catalyst is an Mn_4_Ca complex, which shows most of the typical problems of using DFT. For example, the Mn(III) to Mn(IV) redox energy is very sensitive to the fraction of exact exchange used in hybrid methods like b3lyp. For every change of one percent of the exact exchange, the redox energy changes by one kcal/mol (Siegbahn and Blomberg, 2014). This means that for b3lyp using 10% exact exchange, the redox potential may change by 10 kcal/mol compared to the one using the usual 20%. Since there are three oxidations of Mn(III) in the catalytic cycle the difference becomes 30 kcal/mol from the start to the end. Still, it was possible to make new and certain predictions for water oxidation in PSII, that were confirmed in detail by experiments performed later.

## II. Computational Details

The general computational approach used here has been applied and evaluated for a large number of metallo-enzymes (Blomberg et al., 2014). Density functional calculations are performed on large cluster models of the active sites (170–300 atoms). The active sites in the enzymes discussed here have two or more transition metal ions, and the models include these atoms, plus the first and sometimes also the second shell ligands. As mentioned in the introduction, the b3lyp functional (Becke, 1993) with a varying fraction of exact exchange is used in the present study. A strong argument for using b3lyp as a reference functional, is that this is the hybrid DFT functional with the smallest number of parameters. In fact, the sensitivity of the results for b3lyp is essentially dependent on only one parameter, the amount of exact exchange. Geometries are optimized using a double zeta basis with polarization functions on all second row atoms, and with a few atoms close to the truncations fixed to the X-ray coordinates. The reason not to use a larger basis set for the geometry optimization is based on a large amount of experience gathered during the past three decades, see for example Siegbahn (2001). In fact, an even smaller basis set would probably be accurate enough. More accurate energies for the optimized structures are obtained from single point calculations using a larger basis set, the lacv3p+ basis for the metal ions (Jaguar, 2009), and the large cc-pvtz(-f) basis set for the rest of the atoms. In the recommended use of b3lyp, the fraction of exact exchange has generally been set to 15% (Reiher et al., 2001) in previous studies. Empirical dispersion corrections according to Grimme (Schwabe and Grimme, 2007; Grimme et al., 2010), and solvent effects from the surrounding protein using the self-consistent reaction field (SCRF) approach are included in the energetic results described below. When H_2_ is released and N_2_ becomes bound in nitrogenase, the gain (loss) of translational entropy of about 10 kcal/mol is also included. The inclusion of entropy is very important in these cases but not elsewhere. Zero-point corrections are taken from the Hessians, calculated at the same level as the geometry optimizations. For nitrogenase, these effects were taken from smaller models. The Jaguar 7.9 program (Jaguar, 2009) is used for the nitrogenase geometry optimization and for all the calculations with the larger basis set, and the Gaussian 09 package (Gaussian, 2010) is used for the cytochrome *c* oxidase geometry optimizations and for the Hessian calculations.

The computational procedure described above has been kept as similar as possible to what has been used the past decades. The reason is that a large experience has been gained of this approach, and this knowledge is very useful when the accuracy of the predictions obtained is judged.

## III. Nitrogenase

**Figure 1:**
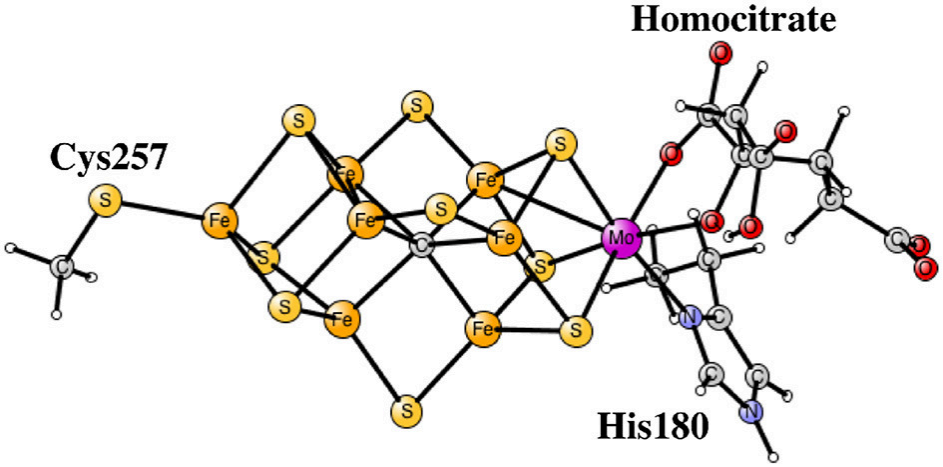
The core structure of the FeMo-cofactor in nitrogenase.

Nitrogenase is the main enzyme in nature that catalyzes nitrogen reduction from the air. The core of its catalytic cofactor is shown Figure 1 (Kim and Rees, 1992; Spatzal et al., 2011). It has seven irons and one molybdenum connected by sulfide bridges. Species containing vanadium and with all-iron exist. A redox potential of –1.6 V, the lowest in nature, is used for the reduction. Quite surprisingly, the oxidation state of the cofactor is not particularly low, which would normally have been expected for a strongly reducing complex. Instead there are four Fe(III) and three Fe(II). Molybdenum is in the Mo(III) state.

Almost all experimental information concerns the ground state before reduction. However, there is one notable exception which is a key for finding the mechanism by DFT model calculations. It has been shown by EPR that the reduced state that activates N_2_, termed E_4_, has two bridging hydrides (Hoffman et al., 2013). These two hydrides were found to be removed as H_2_ in a reductive elimination process, which is directly followed by the binding of N_2_. The process was found to be easily reversible by changing the pressure of hydrogen and nitrogen. This means that the states involved must be nearly isoenergetic.

The experimental suggestion for the structure of E_4_ the central carbon remains unprotonated and has two bridging hydrides (Lukoyanov et al., 2016). Since E_4_ was suggested to appear after four reductions of the experimentally characterized ground state in the catalytic cycle, see Figure 1, there should be two remaining protonations. They were suggested to be on the sulfides. This meant that the redox state for E_4_ should be the same as for the ground state with four Fe(III), a very surprising situation.

**Figure 2:**
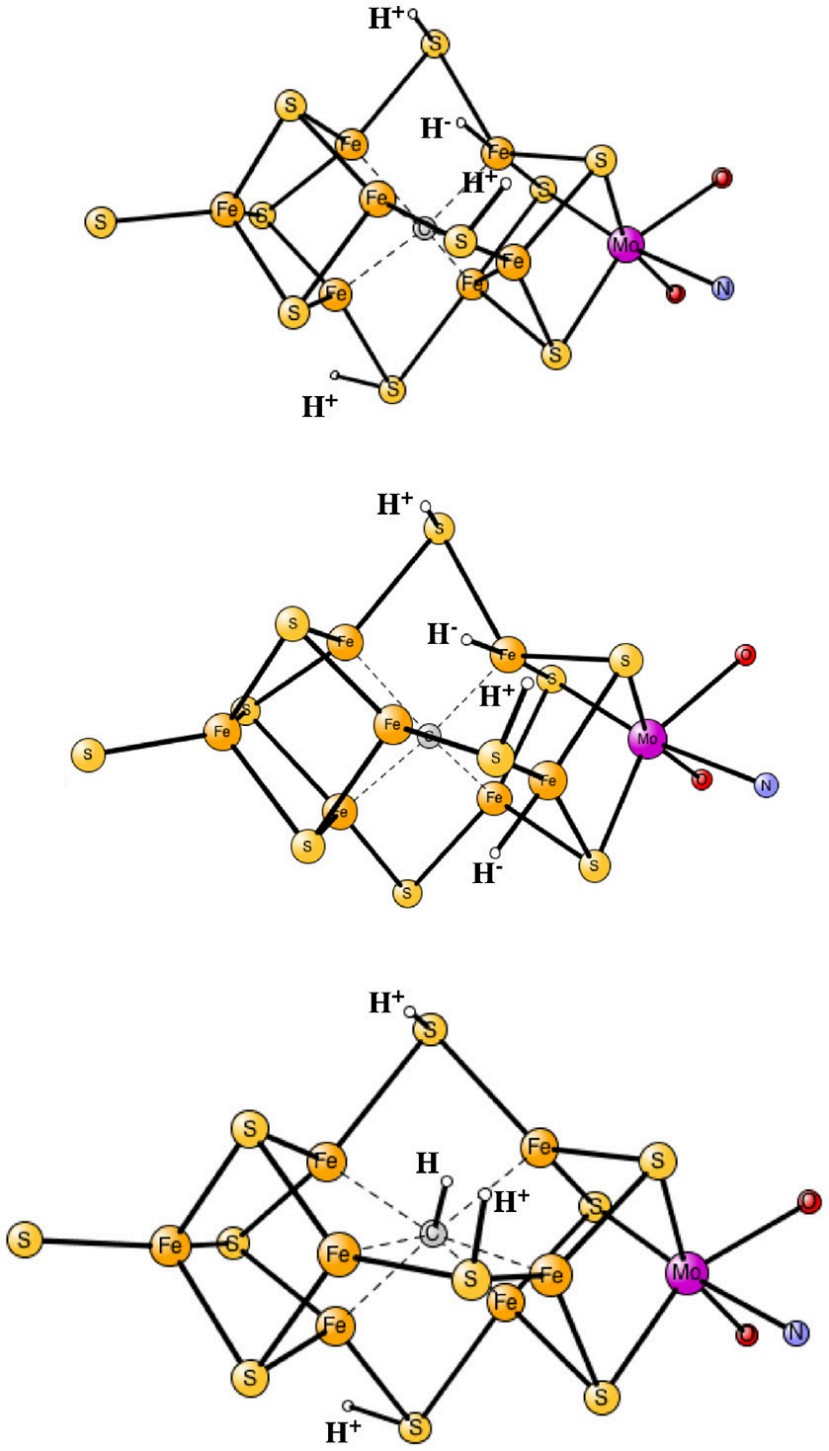
Optimized core structures for the C,H^−^, C,2H^−^, and CH structures.

**Figure 3:**
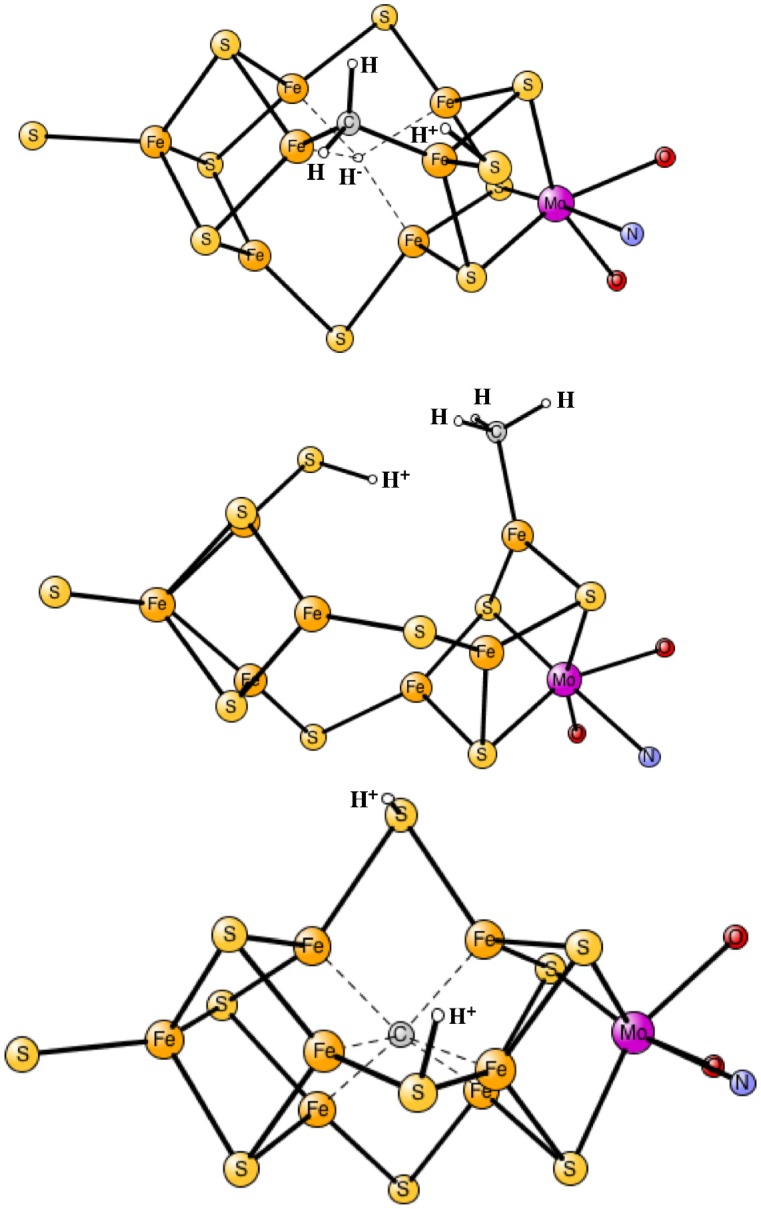
Optimized core structures for the CH_2_,H^−^, CH_3_, and C-2H structures.

The results obtained for nitrogenase after four reductions of the ground state in Figure 1 are shown in Table 1. The corresponding structures are shown in Figures 2, 3. For each functional, the results for six different structures are listed. These structures were taken from the best ones obtained previously, where several hundred structures were compared. They were obtained with 20% exact exchange at the lacvp* level, and were then compared with a large cc-pvtz(-f) basis set at the 15% level. For each functional the geometries were optimized, which turned out to be important. The first structure, termed C,H^−^, has an unprotonated carbon and one hydride. There are three protonations of the sulfides. In the second structure, termed C,2H^−^, one of the protons on the sulfides has moved to a hydride position, so there are two hydrides and two protonated sulfides. This is the one of the six structures that corresponds to the experimentally suggested structure. In the third structure, termed CH, the hydride in the first structure has moved to carbon, and there are still three protonated sulfides remaining. The fourth structure, termed CH_2_,H^−^, has a doubly protonated carbon, one hydride and one protonated sulfide. In the fifth structure, the hydride has moved to carbon to form a CH_3_ group, and there is one protonated sulfide. Finally, in the sixth state, the two hydrides in the second structure has been removed to form a free H_2_ molecule.

**Table 1:** Relative energies (kcal/mol) for states obtained after four reductions from the ground state of nitrogenase, using density functionals with different fractions of exact exchange.

**Structure**	**0%**	**10%**	**15%**	**20%**
C,H^−^	0.0	0.0	0.0	0.0
C,2H^−^	–12.0	+7.2	+14.7	+22.6
CH	+0.1	–9.9	–10.8	–16.1
CH_2_,H^−^	–23.4	–31.7	–34.0	–37.4
CH_3_	–18.1	–30.7	–47.9	–59.2
C - H_2_	–46.0	–36.1	–33.3	–32.0

The main conclusion that can be drawn from the results in Table 1 is that the structure, termed C,2H^−^, does not fit the experimental observations. This is true for all functionals in the table. Already at this point, the important conclusion can be made, that the E_4_ state does not look as suggested experimentally. However, the by far strongest argument against the experimental suggestion comes from the results for the sixth structure. Experimentally, it is known that two hydrides should be removed as H_2_ essentially thermoneutrally. As can be seen in the table, the removal of H_2_ is much too exergonic for all functionals to fit that observation. With 20%, the removal is exergonic by 54.6 (22.6 + 32.0) kcal/mol for 15% exergonic by 48.0 (14.7 + 33.3) kcal/mol, for 10% exergonic by 43.3 kcal/mol and for 0 % exergonic by 34.0 kcal/mol.

It is also found that all functionals prefer a protonated carbon, suggested in previous DFT studies (Siegbahn, 2016). For 20, 15, and 10%, this preference is very large. For 0%, the preference is less pronounced, but it is still there by 11.4 (23.4–12.0) kcal/mol.

It should be added that results with three hydrides are not included in Table 1, but have been done. The reason three hydrides are not in the table is that they can be excluded as possible E_4_ states already from the beginning. It is chemically unreasonable that the first four reductions of the cofactor should lead to an oxidation of the metals rather than a reduction, in particular, since the lowest redox potential of –1.6 V is used. The already high oxidation state of the ground state, with four Fe(III), would be six Fe(III) with three hydrides. The possibility that there should be five Fe(III) and a Mo(IV) is equally unreasonable chemically. Furthermore, for 20, 15 and 10% exchange the structure with three hydrides is very high in energy compared to the other structures. Even for the functional with 0%, the best solution obtained is higher in energy than the CH_2_H^−^ structure by +6.1 kcal/mol. However, that value is somewhat uncertain since the spin-coupling with three hydrides is very different from the other cases, and includes several low-spin-coupled Fe(III). This leads to a very large number of possibilities for the spin-state. Five spin-couplings were tried, all higher in energy than the CH_2_H^−^ structure. They were selected to have the sums of the spins on Fe2-Fe4 opposite to the ones for Fe5-Fe7. Two of them were the ones optimal for the ground state and for the reduced states found previously (Siegbahn, 2016). The last argument against structures with three terminal hydrides is that they are incompatible with the EPR experiments (Hoffman et al., 2013), which clearly show two bridging hydrides.

There are other points of interest in Table 1. For 15 and 20% exchange, the structure with two hydrides are quite high in energy compared to the first structure, by +22.6 and by +14.7 kcal/mol, respectively. Furthermore, they are also terminal, not bridging, hydrides in contradiction to experiments. The energy of this structure is somewhat lower for 10%, but still +7.2 kcal/mol higher than for the starting structure. For 0%, the situation is different. Here the structure with two hydrides is lower than for the first structure by -12.0 kcal/mol, but the two hydrides can still be removed to form H_2_ with a gain of 34.0 kcal/mol, as mentioned above. The lowest state for 20 and 15% is the CH_3_ structure, but for 10 and 0% it is the CH_2_,H^−^ structure, with a margin of –1.0 kcal/mol for 10 % and with –5.3 kcal/mol for 0%.

In summary, there are a few clear conclusions. First, none of the functionals prefers an unprotonated carbon. Also, all functionals bind the hydrides very poorly. To remove the hydrides as H_2_ is strongly exergonic in all cases, by more than 30 kcal/mol, when experiments show that this should be almost thermoneutral. This means that the experimentally suggested structure for E_4_, does not agree with the results for any functional and can be ruled out by a large margin by DFT.

In a very recent paper, a new mechanism for H_2_ release and N_2_ binding in nitrogenase has been suggested (Raugei et al., 2018). A non-hybrid method was used. A mechanism was suggested in which the two hydrides in E_4_ endergonically form a locally bound H_2_ molecule. To avoid the problem with the very large computed exergonicity when H_2_ is released, the key to their mechanism is that this bound H_2_ molecule could only be released with a very high barrier. If the barrier is lower than 18 kcal/mol, there would be no protonation of N_2_, but a high barrier should prevent H_2_ from being released. In their mechanism, the endergonic binding of N_2_ was found to reduce the barrier for releasing H_2_ by a significant amount, and H_2_ can then be released. There are many bound H_2_ complexes in the literature, but none of them behaves like suggested in Ref. (Raugei et al., 2018). In all the published cases there is at most a weakly bound H_2_, which can be released without additional barriers apart from the endergonicity. A search for a bound H_2_ was initiated in the present study using 0% exchange (non-hybrid), and a picture is obtained which is very similar to the ones previously published in the literature. A weak local minimum for a bound H_2_ is obtained and releasing it from that minimum goes over at most a very small barrier, less than 5 kcal/mol. The release is quite exergonic including a gain of entropy of about 10 kcal/mol.

**Figure 4:**
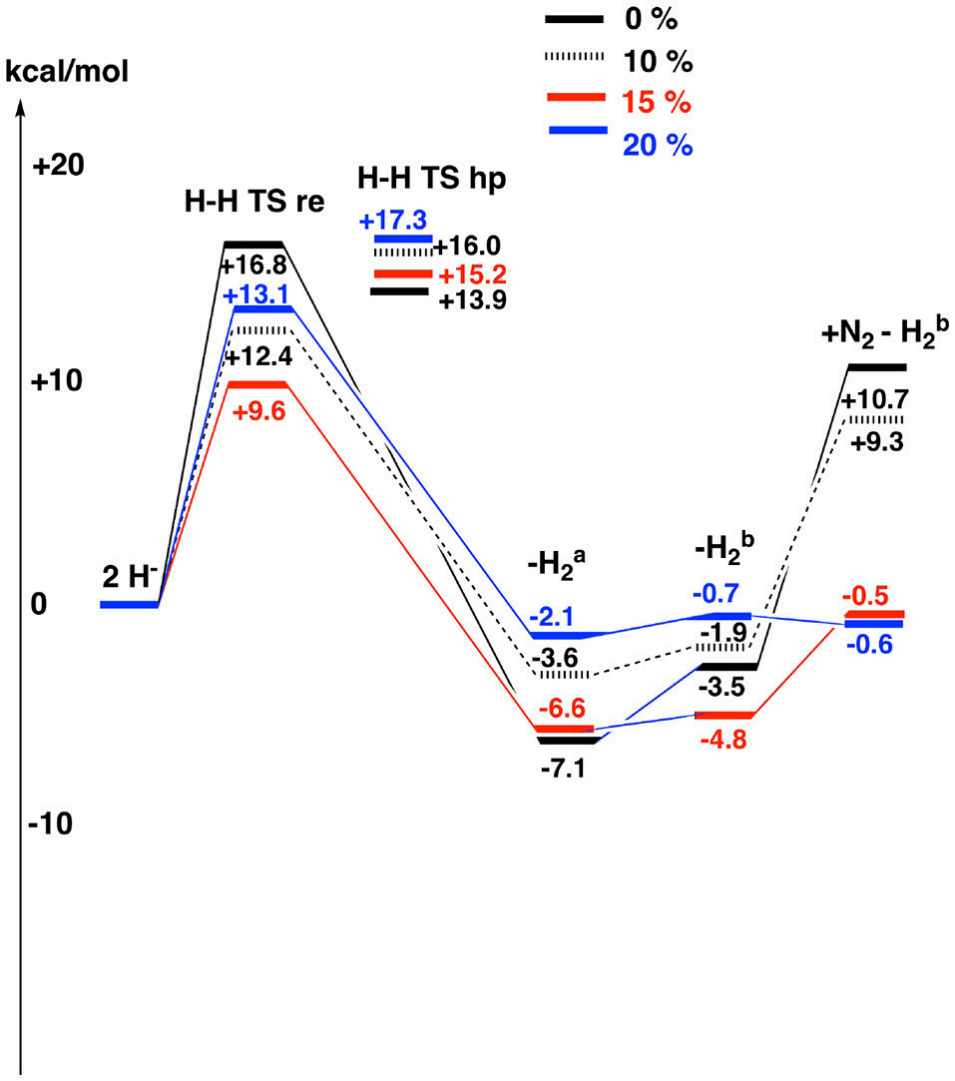
The results for the theoretically suggested E_4_ structures are shown for different fractions of exact exchange used , see also Table 2.

Since DFT rules out an unprotonated carbon structure, another structure has to be found for E_4_. If no other structure can be found, the conclusion must be that no version of DFT can handle nitrogenase, a very unlikely scenario. It has been suggested in previous DFT studies that the lowest energy structure in Table 1, is merely a starting structure for catalysis (Siegbahn, 2016). The first four reductions of the ground state structure in Figure 1 would then be just an initial activation process, done only once, before catalysis starts leading to a new E_0_ state. Following that suggestion, four additional reductions would lead to the actual E_4_ structures. The results for the E_4_ structures, determined in this way, are shown in Table 2 for the same four functionals as discussed above. The results are also displayed in Figure 4. The results for six structures are shown, following the conclusions of the EPR experiments. The first entry, termed 2H^−^, has two hydrides, a CH_3_ ligand and three protonated sulfurs, altogether eight protonations of the cofactor. The second entry, termed “H-H re TS” shows the barrier for the re mechanism (reductive elimination) of two hydrides to form H_2_. The third entry, termed “H-H hp TS,” shows the barrier for the hp (hydride, proton) mechanism, where one hydride and one proton form H_2_. In the fourth entry, termed “- ${\text{H}}_{2}^{a}$,” the two hydrides have been removed to form a free hydrogen molecule. The fifth entry, termed “- ${\text{H}}_{2}^{b}$”, differs from the second one by a rotation of the homocitrate ligand. This rotation was found in earlier studies (Siegbahn, 2018) to be required for binding N_2_. In the final sixth entry, termed “+ N_2_–${\text{H}}_{2}^{b}$,” N_2_ binds to the fifth structure.

**Table 2:** Relative energies (kcal/mol) for states obtained after eight reductions from the ground state of nitrogenase, using density functionals with different fractions of exact exchange.

**Structure**	**0%**	**10%**	**15%**	**20%**
2H^−^	0.0	0.0	0.0	0.0
H−H TS re	+16.8	+12.4	+9.6	+13.1
H−H TS hp	+13.9	+16.0	+15.2	+17.3
− H_2_^a^	–7.1	–3.6	–6.6	–2.1
− H_2_^b^	–3.5	–1.9	–4.8	–0.7
+ N_2_ - H_2_^b^	+10.7	+9.3	–0.5	–0.6

a *H_2_ is removed with homocitrate non-rotated*.

b *H_2_ is removed with homocitrate rotated*.

The results for the functional with 20% agree very well with the EPR experiments. H_2_ can be removed with a very small energy gain of –2.1 kcal/mol. The barrier for H_2_ elimination using the re mechanism is 13.1 kcal/mol, well within the range required by experiments. Even more importantly, the barrier using the hp mechanism of 17.3 kcal/mol is 4.2 kcal/mol higher than for the re mechanism, leading to the required N_2_ activation rather than H_2_ production. The following homocitrate rotation is only uphill by +1.4 kcal/mol. In the next step, N_2_ binds exergonically to the third structure by –0.1 kcal/mol. This means that the release of H_2_ and the binding of N_2_ should be easily reversible as observed experimentally. There is a minor discrepancy, since there should be some driving force (exergonicity) for this process, but the error is small for such a complicated process. Going to 15%, the discrepancy is somewhat larger. The release of H_2_ is exergonic by -6.6 kcal/mol and N_2_ binding is endergonic by +4.3 kcal/mol. However, the discrepancy to experiments is not alarming. Again, the barrier eliminating H_2_ by the re mechanism is preferred over the one using the hp mechanism, now by 6.2 kcal/mol. For the functional with 10%, the discrepancy to experiments is significantly increased. Most noteworthy, the binding of N_2_ is now endergonic by + 11.2 (9.3 + 1.9) kcal/mol. However, the barrier for the re mechanism is still lower than the one for the hp mechanism. Finally, for the functional with 0%, the discrepancy to experiments increases further. For example, the binding of N_2_ is now endergonic by +14.2 kcal/mol. Furthermore, the barrier for the hp mechanism is now lower than for the re mechanism, leading to production of H_2_ rather than protonation of N_2_.

## IV. Cytochrome *C* Oxidase

The membrane bound enzyme cytochrome *c* oxidase (C*c*O) catalyzes the reduction of molecular oxygen to water as the last step in the respiratory chain in aerobic organisms. The chemistry occurs in an active site consisting of a high-spin heme group, a copper complex, labeled Cu_*B*_, and a redox active tyrosine, referred to as the binuclear center (BNC). The electrons are delivered to the BNC from a reduced cytochrome *c*, located on the positive side of the membrane. The protons are transferred to the BNC from bulk water on the negative side of the membrane. Molecular oxygen binds to the reduced BNC, with heme-Fe(II), Cu_*B*_(I) and TyrOH. The O-O bond is cleaved in a single reaction step, yielding a four electron oxidized BNC, with heme-Fe(IV)=O, Cu_*B*_(II)OH and a neutral TyrO-radical (Proshlyakov et al., 1998; Fabian et al., 1999). The rest of the catalytic cycle consists of four reduction steps, each taking up one electron and one proton to the BNC, leading back to the reduced state with two new water molecules. The overall energetics of the reduction process is obtained from the difference in reduction potential between the electron donor and the acceptor, molecular oxygen. With cytochrome *c* as the electron donor, reduction of one oxygen molecule becomes exergonic by 50.7 kcal/mol (2.2 eV) (Brzezinski, 2004). A significant part of this free energy is conserved as an electrochemical gradient across the membrane, which in turn is used by another enzyme, ATP-synthase, to produce ATP, the energy currency of the cells. Two processes contribute to the gradient buildup, one is the electrogenic chemistry (taking the electrons and the protons from opposite sides of the membrane), and the other is the so called proton pumping, which means that the chemistry is coupled to proton transfer across the entire membrane. The largest group of C*c*O:s are known to pump one proton per electron, i.e., four protons per oxygen molecule (Brzezinski and Gennis, 2008; Kaila et al., 2010). The mechanism for the proton pumping, i.e., how to couple the transfer of one electron to the active site with the uptake of two protons from the negative side of the membrane, is still under debate (Rich, 2017).

The process of oxygen reduction in C*c*O has been studied in detail using density functional theory. A mechanism for the O-O bond cleavage step was suggested based on computational results at an early stage (Blomberg and Siegbahn, 2006, 2010), and it was later confirmed in a combined experimental and computational study (Poiana et al., 2017). Another result from the computational studies concerns the mechanism of proton pumping, for which it has been suggested that the redox active tyrosine in the active site plays an essential role (Blomberg, 2016). Experimental support for the suggested pumping mechanism is that an active site tyrosine is conserved in all families of C*c*O's (Hemp et al., 2006). To understand the proton pumping in C*c*O it is essential to know the energetics of the individual reduction steps in the catalytic cycle, which depends on the reduction potential of the active site cofactor that is reduced in each particular step. Experimental investigations have indicated that the four different active site reduction potentials involved are quite different, and that only two of them seem to be large enough (about 0.8 V) to afford proton pumping (Wikström and Morgan, 1992; Kaila et al., 2010). Also the BNC potentials have been studied computationally, with result that partly differ from the experimental measurements (Blomberg and Siegbahn, 2015a), see further below. In all these computational studies the b3lyp type of functional was used as described above in Computational details.

**Figure 5:**
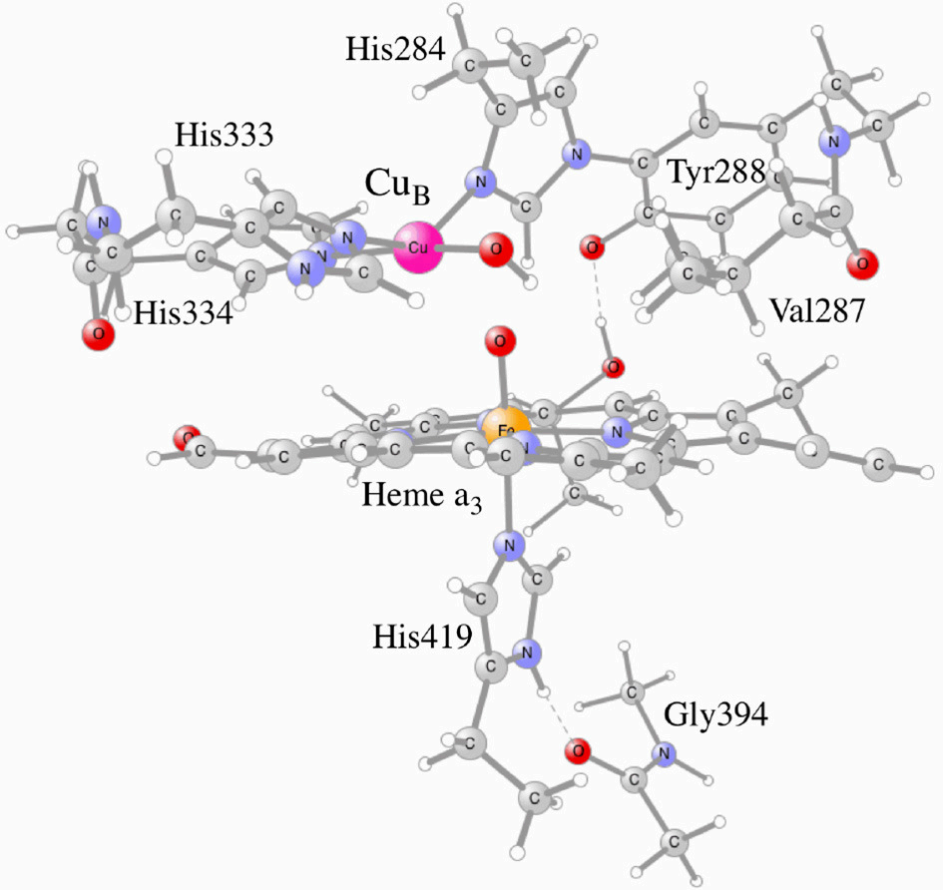
The C*c*O model used in the calculations, showing the active site cofactors in the four electron oxidized state: Cu_*B*_(II)-OH, TyrO(288)-radical and heme a_3_-Fe(IV)=O.

To further test the reliability of the results obtained for C*c*O, it was decided to systematically investigate the calculated proton coupled reduction potentials for the active site cofactors by varying the fraction of exact exchange in the b3lyp potential, using the model shown in Figure 5. It is noted that each of the proton coupled reduction potentials corresponds to the formation of a new O-H bond in the active site. The individual reduction potentials can therefore be estimated from the strengths of the different O-H bonds. The strength of an O-H bond is not sensitive to a distant surrounding, since the charge is not changed by the addition of a (H^+^,e^−^)-couple, which means that reasonably sized models (150–200 atoms) can be used in the calculations. For each functional (different percentage of exact exchange) new structures are optimized, and since the most recently used procedure for C*c*O has been to optimize the structures with dispersion, the D3 dispersion correction with parameters from the original b3lyp-D3 functional (Grimme et al., 2010) was used in both geometry optimizations and energy calculations. Since results for the functional with 15% were also obtained using structures optimized with 20% (not reported) it could be noted that the final energetics differs only slightly (<2 kcal/mol) from the ones reported below.

To estimate the energetics of the reduction steps of the C*c*O catalytic cycle, the sum of the energy of an electron transferred from cytochrome *c* plus a proton transferred from bulk water is needed. This energy is parameterized to reproduce the experimental results for the overall reaction (50.7 kcal/mol, see above). By subtracting the parameterized energy from each O-H bond strength, the exergonicity relative to the cytochrome *c* donor is obtained, and by comparing to the midpoint potential of cytochrome *c* (0.25 V) the midpoint potential can be estimated for each cofactor. The first result to be discussed concerns the proton coupled reduction potential for Cu_*B*_, for which the experimental measurements have given low values, 0.2–0.4 V (Jancura et al., 2006; Brand et al., 2007; Vilhjámsdóttir et al., 2018). In contrast, the previous calculations indicate a much higher value (0.9–1.0 V) during catalytic turnover (Blomberg and Siegbahn, 2015a), and an explanation for the low experimental values has been suggested based on the computational results (Blomberg and Siegbahn, 2015b). As can be seen in Table 3, all functionals support a large reduction potential for Cu_*B*_, with results that vary only slightly and in a somewhat irregular way with the fraction of exact exchange in the functional, 0.97-1.19 V. Interestingly, using a small model complex it was also shown that the b3lyp^*^ (15%) results for the O-H bond strength in Cu_*B*_(I)OH_2_ agrees to within one kcal/mol with CCSD(T) results (Blomberg and Siegbahn, 2015a).

**Table 3:** Calculated energetics as a function of the amount of exact exchange for the reduction of Cu_*B*_ (taking up one electron and one proton) in the catalytic cycle of cytochrome *c* oxidase.

**Reduction step**	**0%**	**10%**	**15%**	**20%**	**exp^a^**
**Cu**_*B*_**(II)OH**→**Cu**_*B*_**(I)OH**_2_					
ΔH(O-H) (kcal/mol)	89.0	85.2	84.3	86.7	
ΔG^b^ (kcal/mol)	–21.7	–17.5	–16.5	–18.8	
E_*m*, 7_ (V)	1.19	1.01	0.97	1.07	0.4+

a * From Kaila et al. (2010). Although the experimental measurements have given low values, below 0.4 V, it has been suggested based on experiment that during turnover the Cu_B_ potential actually is higher than the measured values (Kaila et al., 2010)*.

b *Exergonicity relative to cytochrome c, with a reduction potential of 0.25 V*.

As mentioned above, experiments have shown that two of the active site reduction potentials are about 0.8 V (Kaila et al., 2010), which is large enough for proton pumping in the corresponding reduction steps. These two steps concern reduction of the tyrosyl radical and heme-Fe(IV)=O, respectively (Kaila et al., 2010). The calculated results for these two cofactors using the different functionals are reported in Table 4. For the tyrosyl radical the experimental value is 0.82 V, and all functionals with a fraction exact exchange (10–20%) are in reasonable agreement with experiment, 0.73–0.86 V. Again, the reduction potential does not vary very much with the exact exchange, and even the functional without exact exchange (0%) is not too far off, with a value of 0.60V. For the heme-Fe(IV)=O reduction the situation is quite different. The experimental value is 0.76 V (Kaila et al., 2010), and here the functional with 15% gives the best agreement, with a value of 0.68 V. The result with 20%, 1.01 V, is also in reasonable agreement with experiment. The result without exact exchange (0%) disagrees qualitatively with experiment, the calculated value is –0.22 V, and also with only 10% exchange the calculated value, 0.45 V, is quite far from the experimental value. An important factor for the strong variation of this reduction potential is that there is a change of spin coupling in the heme-Fe(IV)=O → heme-Fe(III)OH transition, where iron is low-spin coupled in heme-Fe(IV)=O and high-spin coupled in heme-Fe(III)OH.

**Table 4:** Calculated energetics as a function of the amount of exact exchange for two of the four reduction steps (taking up one electron and one proton) in the catalytic cycle of cytochrome *c* oxidase.

**Reduction step**	**0%**	**10%**	**15%**	**20%**	**exp^a^**
**TyrO^•^ → TyrOH**					
ΔH(O−H) (kcal/mol)	75.4	78.7	81.1	82.0	
ΔG^b^ (kcal/mol)	–8.1	–11.0	–13.3	–14.1	
E_*m*, 7_ (V)	0.60	0.73	0.83	0.86	0.82
**heme-Fe(IV)=O**→**heme-Fe(III)OH**					
ΔH(O−H) (kcal/mol)	56.4	72.4	77.7	85.5	
ΔG^b^ (kcal/mol)	+10.9	–4.7	–9.9	–17.6	
E_*m*, 7_ (V)	–0.22	0.45	0.68	1.01	0.76

a *From Kaila et al. (2010)*.

b *Exergonicity relative cytochrome c, with a reduction potential of 0.25 V*.

## V. Conclusions

By using the present systematic approach of DFT, a few major conclusions can be drawn for the nitrogenase mechanism. In Table 1, the results are given for different E_4_ structures obtained after four reductions of the ground state, as has been suggested experimentally. The most important result here is that no functional gives results consistent with the conclusions of the experimental EPR study (Hoffman et al., 2013). In particular all functionals give a very large exergonicity for releasing H_2_. This means that the experimentally suggested structure can not be supported by any DFT functional. The discrepancy to experiments is very large, independent of fraction of exact exchange used. Even the non-hybrid method (0%), gives very poorly bound hydrides, which can be removed with a gain of 34 kcal/mol, when experiments indicate that the release should be nearly thermoneutral. In contrast, the theoretically suggested structure for E_4_, obtained after eight reductions of the ground state, shows a much more reasonable agreement with the experimental EPR information, in particular, with 20 and 15% exchange. Since DFT indeed found a structure that agrees well with what is known for the E_4_ state, a conclusion that DFT totally fails for nitrogenase is very farfetched. If that would have been the case, it would be the first example among the many redox reactions studied, that would show that behavior (Blomberg et al., 2014). If no structure would have been found that agreed with experimental information for E_4_, the conclusion could have been different. A very recent study with many different functionals did not find any functional that gave a preference for the experimental structure (Cao et al., 2018), in agreement with the prediction made here.

For C*c*O, the calculations show that the active site cofactor Cu_*B*_ has a large midpoint potential, about 1 V, a result obtained regardless of the fraction of exact exchange in the functional. This is in contrast to the much lower potentials obtained in experimental measurements, but in agreement with experimental observations on proton pumping. For another active site cofactor, heme-Fe(IV)=O, the functional with 15% exact exchange gives the best agreement with experimental observations for the midpoint potential, and the functional without exact exchange gives qualitatively wrong results.

In summary, the results for the different functionals show that the best agreement with experiments, for both nitrogenase and cytochrome *c* oxidase, is obtained with 15–20% exact exchange in the functional. 10% exact exchange gives results slightly worse, and the use of a non-hybrid functional (0% exact exchange) gives qualitatively wrong potential surfaces in both cases. These results are in line with experience gathered during the past two decades (Blomberg et al., 2014). This experience can be used in two ways. It could either be used to calibrate the exact exchange fraction based on some well-known experimental fact for that system. Or, the difference between the results for 15 and 20% could be used as an estimate of the error in the calculations. The latter approach has mainly been used in our previous studies.

## Author Contributions

PS wrote the paper and did nitrogenase calculations. MB wrote the paper and did cytochrome *c* oxidase calculations.

### Conflict of Interest Statement

The authors declare that the research was conducted in the absence of any commercial or financial relationships that could be construed as a potential conflict of interest.
